# IS THE PATH EASIER ON THE BRIGHTER SIDE? OPTIMISM AND DAILY STRESS PROCESSES ACROSS 16 YEARS

**DOI:** 10.1093/geroni/igz038.2998

**Published:** 2019-11-08

**Authors:** Lewina O Lee, Avron Spiro, Daniel K Mroczek, Laura D Kubzansky

**Affiliations:** 1 Boston University School of Medicine, Boston, Massachusetts, United States; 2 VA Boston Healthcare System, Boston, Massachusetts, United States; 3 Northwestern University Feinberg School Of Medicine and Weinberg College of Arts & Sciences, Chicago, Illinois, United States; 4 Harvard T.H. Chan School of Public Health, Boston, Massachusetts, United States

## Abstract

Accumulating evidence supports optimism as a health asset, yet little is known about underlying pathways. This study evaluates the long-term influence of optimism on exposure and affective reactivity to daily stressors. The sample comprised 233 community-dwelling men who completed a validated measure of optimism in 1986 (age: M=59, SD=6), and participated in up to three 8-day daily diary studies between 2002-10. Daily stressor occurrence, end-of-day positive affect (PA) and negative affect (NA) were assessed nightly in the diary studies. Results from multilevel structural equation modeling showed that optimism was unrelated to affective stress reactivity. However, higher optimism preceded lower overall NA and higher overall PA. Lower daily stressor exposure mediated the association from optimism to lower NA (indirect effect: B=-0.26, 95% Bayesian CI: -0.48, -0.09), but it did not account for the optimism-PA association. Our findings add to knowledge on pathways by which optimism promotes affective well-being in old age.

